# Facile Synthesis of Porous Polymer Using Biomass Polyphenol Source for Highly Efficient Separation of Cs^+^ from Aqueous Solution

**DOI:** 10.1038/s41598-020-65099-6

**Published:** 2020-05-19

**Authors:** Shangqing Chen, Jiayin Hu, Yafei Guo, Tianlong Deng

**Affiliations:** Tianjin Key Laboratory of Brine Chemical Engineering and Resource Eco-utilization, College of Chemical Engineering and Materials Science, Tianjin University of Science and Technology, Tianjin, 300457 P.R. China

**Keywords:** Environmental sciences, Materials science

## Abstract

In this work, a series of polyphenol porous polymers were derived from biomass polyphenols *via* a facile azo-coupling method. The structure and morphologies of the polymer were characterized by BET, TEM, SEM, XRD, TGA and FT-IR techniques. Batch experiments demonstrated their potentialities for adsorptive separation of Cs^+^ from aqueous solution. Among them, porous polymers prepared with gallic acid as starting material (GAPP) could adsorb Cs^+^ at wide pH value range effectively, and the optimal adsorption capacity was up to 163.6 mg/g, placing it at top material for Cs^+^ adsorption. GAPP exhibited significantly high adsorption performance toward Cs^+^ compared to Na^+^ and K^+^, making it possible in selective removal of Cs^+^ from ground water in presence of co-existing competitive ions. Moreover, the Cs-laden GAPP could be facilely eluted and reused in consecutive adsorption-desorption processes. As a result, we hope this work could provide ideas about the potential utilization of biomass polyphenol in environmental remediation.

## Introduction

Growing concern about the remediation of radioactive waste generated from nuclear power plants and unforeseen accidents have been given worldwide for the sake of human health and environmental issues^1–4^. Among them, radio-isotope ^137^Cs is considered as the most hazardous nuclide due to the serious gamma radiation, long half-life as well as high solubility being an alkaline element^5,6^. The generation of hazardous ^137^Cs by nuclear waste and unforeseen nuclear plant accidents has seriously threatened the global environment and human health. In addition, long-term exposure to ^137^Cs-contanining wastewater would lead to horrible diseases such as cancer, leukemia and genetic disorder^7^. For example, the accidents occurred at Chernobyl in 1986 and Fukushima in 2011 severely impacted the local environments, and the surrounding areas are still classified as dangerous regions due to the leakage and serious emission of ^137^Cs and other radio-isotopes^8,9^. Therefore, effective techniques for the decontamination of radioactive Cs^+^ from wastewater are indispensable and highly desirable.

So far, considerable efforts have been made to explore available methodologies for hazardous Cs^+^ removal from radioactive wastewater, such as liquid-liquid solvent extraction, chemical precipitation, electrochemical techniques and adsorption process^10–12^. Taking disposal cost and removal efficiency into consideration, adsorption is considered as one of the most effective and clean techniques, and has been widely used in Cs^+^ removal. To date, Prussian blue (PB) analogues^12,13^, titanate nanomaterials^14^, metal oxides and sulfides^15^, natural zeolites^16^, ammonium molybdophosphate^17,18^ and other adsorbents^19^ were developed and used for the removal of Cs^+^ from radioactive effluents. Unfortunately, they still suffered from several problems, such as considerable preparation cost, unsatisfactory adsorption performance and insufficient stability, thus majority of the adsorbents aforementioned were not environmental friendly, economically and industrially attractive. Therefore, the development of cost-effective, durable and effective Cs^+^ adsorption materials are still particularly urgent.

More recently, it has been reported that resorcinol formaldehyde (RF) resin had a favorable affinity for Cs^+^ due to the presence of phenolic hydroxyl groups^20^, and Yang *et al*. demonstrated the phenolic hydroxyl exchange mechanism for Cs^+^^21^. After that, easily available biomass materials with abundant polyphenolic groups have been used for Cs^+^ adsorption. For example, Gurung and co-workers developed cross-linked persimmon tannin and tea leaves for Cs^+^ removal, and it showed favorable selectivity and removal efficiency^22^. Pangeni *et al*. synthesized a kind of cross-linked persimmon waste to uptake Cs^+^ from wastewater and obtained an adsorption capacity of 71.8 mg/g^23^. Although the adsorbents prepared by cross-linking method showed good adsorption performance for Cs^+^, however, the cross-linked persimmon tannin, tea leaves and persimmon waste adsorbents were difficult to recycle and reused, which were not economically attractive. Thus, further work are strongly desired to solve these problems.

Porous polymers (PPs) have been regarded as a kind of promising and unique materials, which have received considerable interests^24,25^. PPs can be designed to be with multiple functionalities by introducing various functional monomers, which endows them with novel properties and extensive applications in adsorption^26,27^, separation^28,29^, catalysis^30^, hydrogen storage^31^ and so on. To the best of our knowledge, there were negligible researches on the synthesis and application of PPs using biomass polyphenols sources on Cs^+^ removal.

Herein, a series of polyphenol porous polymers were derived from biomass polyphenols sources *via* a facile azo-coupling method and provided a range of possibilities for Cs^+^ separation from aqueous solution. Due to the porous structures functionalized by biomass polyphenols, the gallic acid-based porous polymer (GAPP) exhibited exceptionally adsorption performance for Cs^+^ (163.6 mg/g). Moreover, this low-cost and environmentally friendly GAPP was robust in either acidic or basic solution (pH value range 2–12), and showed stable performance in consecutive adsorption-desorption experiments.

## Results and Discussion

### Characterization

**Figure 1:**
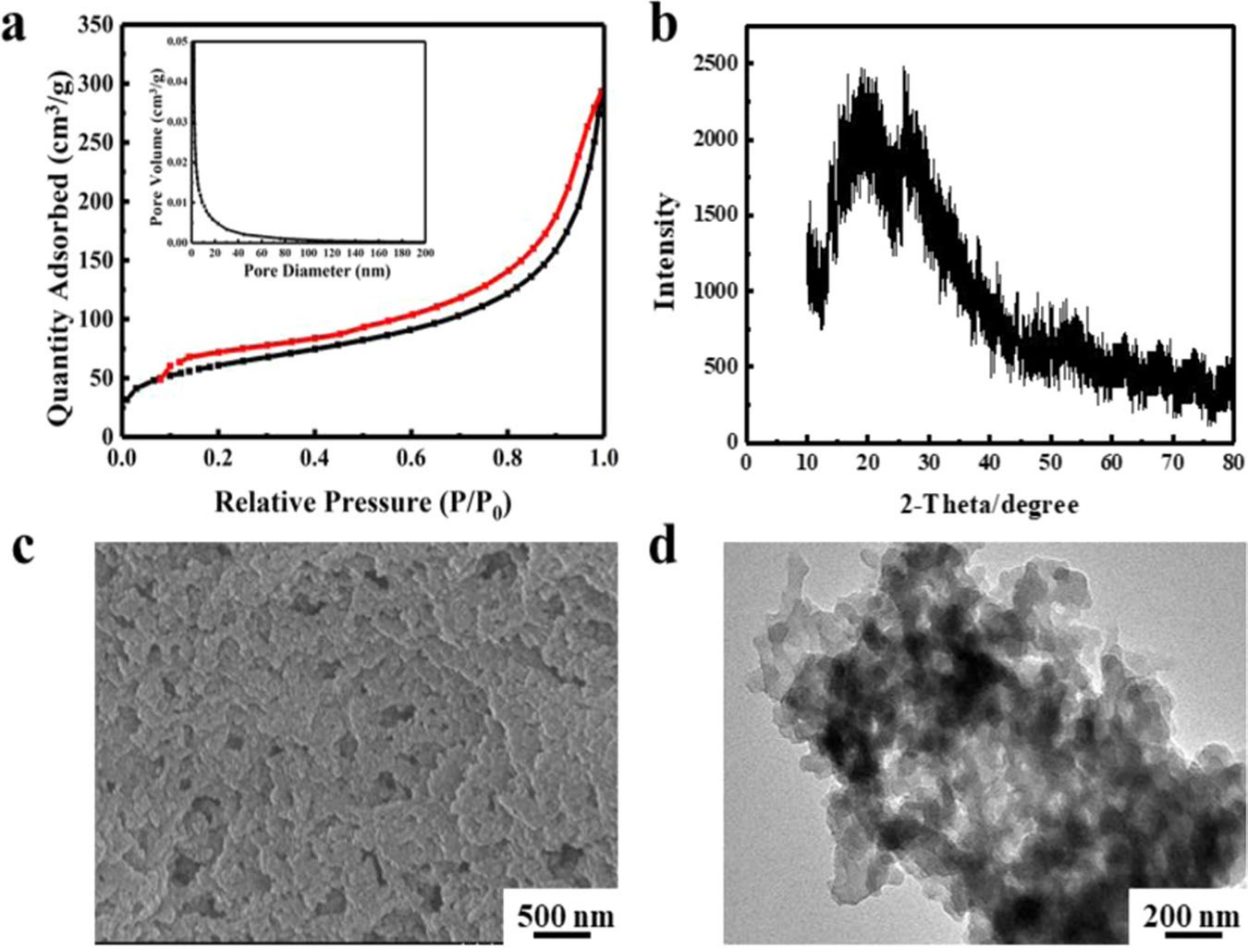
Characterization for the prepared GAPP: (**a**) N_2_ adsorption-desorption isotherm, pore size distribution (inset); (**b**) XRD pattern; (**c**) SEM image; (**d**) TEM image.

**Figure 2:**
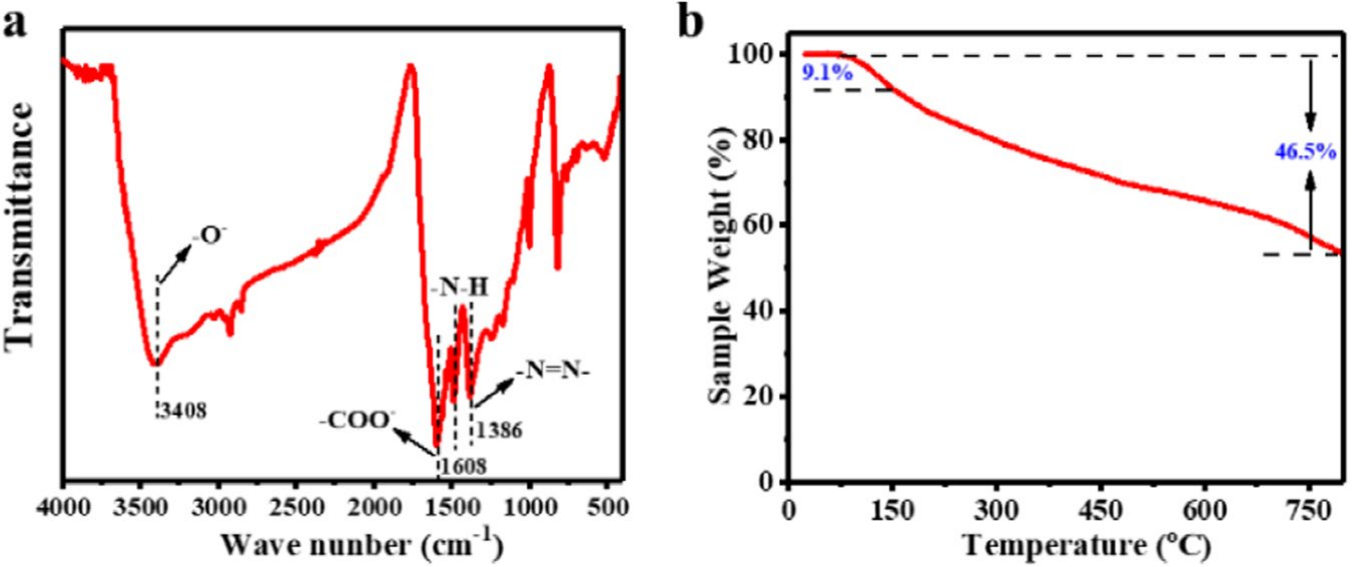
FT-IR spectrum (**a**) and thermogravimetric analysis (**b**) of GAPP.

The N_2_ adsorption-desorption isotherm (Fig. 1a) showed the porous structure of GAPP with a wide pore-size distribution (the inset in Fig. 1a, measured by Barret-Joyner-Halenda (BJH) method), and the specific surface area of 220 m^2^/g and pore volume of 0.42 cm^3^/g were calculated, respectively^32,33^. Due to the incomplete desorption in low pressure, there was a desorption hysteresis in N_2_ isotherm. According to the isotherm classification system (IUPAC), the isotherm classification of GAPP was tend to Type II. Further SEM (Fig. 1c) and TEM (Fig. 1d) images also confirmed the porosity of GAPP, which was agreed with the results from N_2_ adsorption. Additionally, XRD pattern (Fig. 1b) exhibited a broad peak, indicating the low crystallinity and amorphous structure of GAPP. FT-IR spectroscopy (Fig. 2a) showed that the IR frequencies appeared at 1608 and 3408 cm^−1^ indicated the presence of –COO^−^ and –O^−^ ^34^, and the bands at 1200 and 1400 cm^−1^ were attributed to the presence of the azo group, which proved the successful formation of the -N = N- band in the prepared GAPP^29,30^. The thermogravimetry analysis in Fig. 2b indicated the stable structure even in relatively high temperature (weight loss was less than 9.1% within 150 °C).

### Adsorption performance

**Figure 3:**
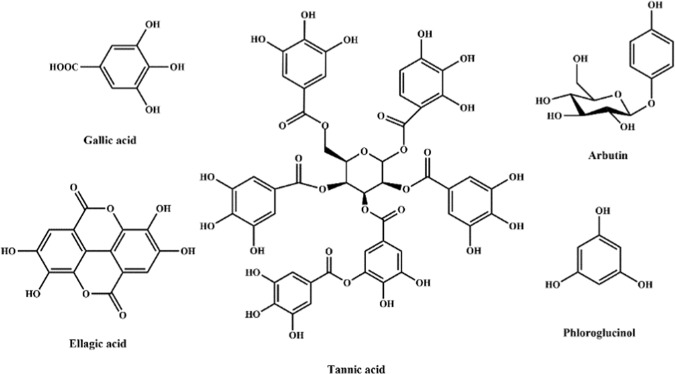
The structures of biomass polyphenols used in this work.

**Figure 4:**
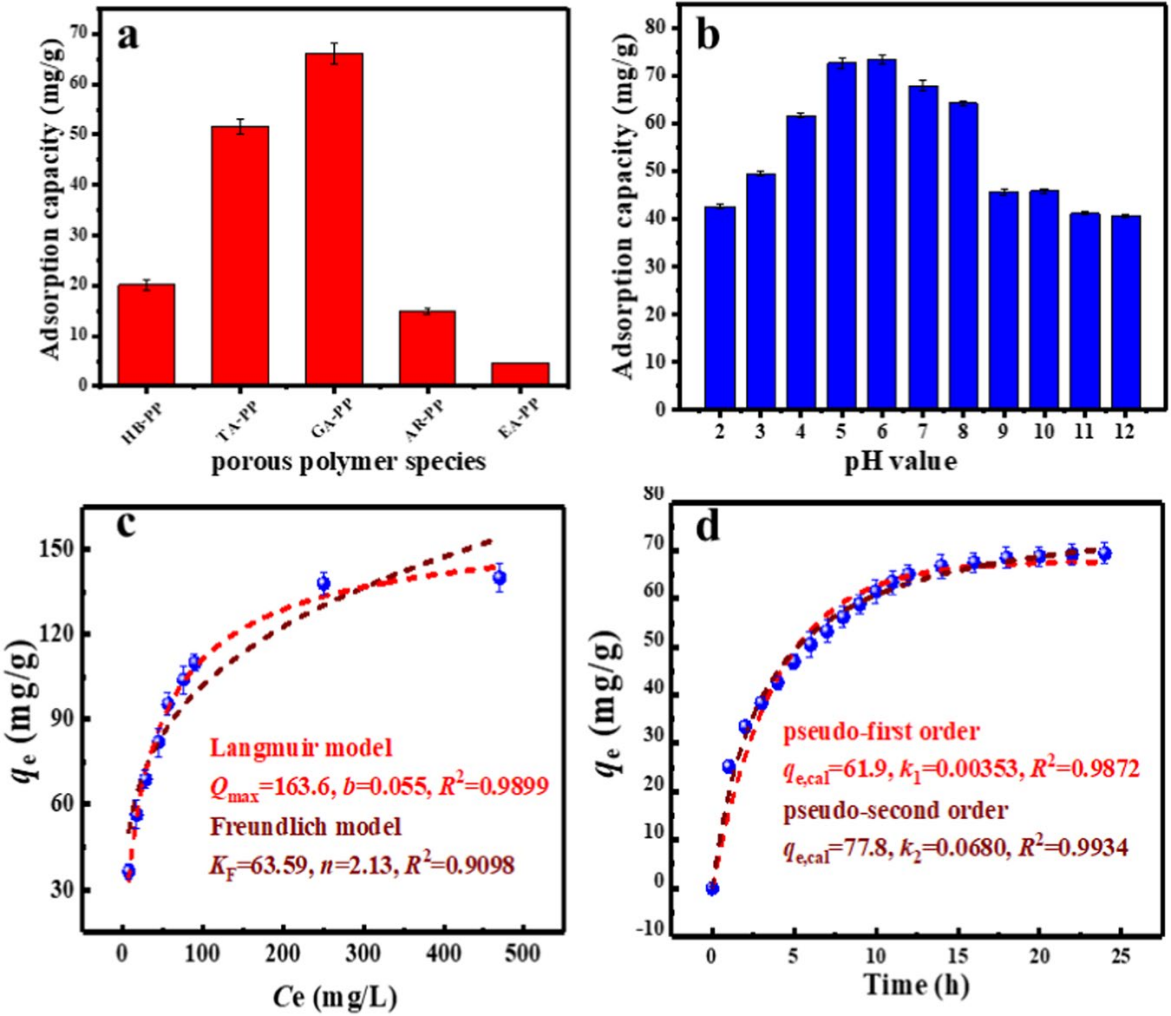
(**a**) Adsorption capacities of different PPs for Cs^+^ (*C*_0_ = 100 mg/L, *m*/*V* = 1 g/L, pH = 6.0, *t* = 24 h, *T* = 298.15 K); (**b**) Effect of pH value on Cs^+^ adsorption by GAPP (*C*_0_ = 100 mg/L, *m*/*V* = 1 g/L, *t* = 24 h, *T* = 298.15 K); (**c**) Effect of Cs^+^ concentration and adsorption isotherm on adsorption (*C*_0_ = 0–500 mg/L, *m*/*V* = 1 g/L, pH = 6.0, *t* = 24 h, *T* = 298.15 K); (**d**) Effect of contact time and adsorption kinetic on adsorption (*C*_0_ = 100 mg/L, *m*/*V* = 1 g/L, pH = 6.0, *T* = 298.15 K).

A series of polyphenol porous polymers (PPs) were prepared using biomass polyphenols sources including phloroglucinol, tannic acid, arbutin and ellagic acid (Fig. 3) as starting materials, which were derived from biomass, such as persimmon, tea leaves, nut and so on. Their potentialities for Cs^+^ adsorption were evaluated by batch experiments and the results are present in Fig. 4a. It was found that PPs obtained with different hydroxyl-containing starting materials were all efficient for Cs^+^ adsorption. Especially, GAPP derived from gallic acid that had plentiful functional phenolic groups and weaker steric hindrance, showed the best adsorption performance for Cs^+^ than other PPs, indicating the great performance of gallic acid on Cs^+^ adsorption. Therefore, GAPP was selected for further adsorption experiments hereafter.

### Effect of pH value

Firstly, the influence of pH value on the adsorption by GAPP was investigated, and the experiments were proposed with Cs^+^ concentration of 100 mg/L and pH ranged from 2–12. As Fig. 4b showed, the GAPP was highly durable and could adsorb Cs^+^ within pH ranging 2–12. Moreover, the *q*_e_ increased firstly when pH value increased from 2–6, and then decreased at pH value larger than 7.

**Scheme 1:**
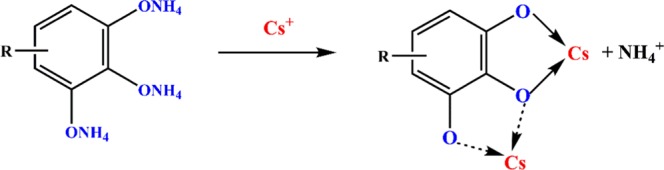
The proposed adsorption mechanism of GAPP with Cs^+^ (dotted arrow: electrostatic interaction; solid arrow: coordination interaction).

The FT-IR data of GAPP before and after Cs^+^ were compared in the Supporting Information (Fig. S1). It was found that the characteristic peak of -O- shifted to a slightly lower wavenumber after Cs^+^ adsorption due to the coordination between -O- and the adsorbed Cs^+^. This shifting was caused by the changes in force constants of the bonds as well as geometry of the O atoms after coordination^22,23^. Therefore, According to the experimental results and FT-IR spectra, the adsorption mechanism is proposed as Scheme 1.

When pH values were low, -ONH_4_ group was likely to retain protons, thus resulting in insufficient adsorption sites and low adsorption capacity of GAPP for Cs^+^. With the increase of pH values, -ONH_4_ group was gradually transformed into -O^-^. It could effectively interact with the polyphenolic groups of GAPP to form chelating cyclic metallacomplex, and the optimal adsorption capacity was achieved at pH 6.0. When pH values kept increasing, the adsorption capacity decreased because of much stronger ionic strength^35^. Moreover, there was electrostatic interaction between Cs^+^ and –O^−^ group. Therefore, the adsorption mechanism was considered to be coordination interaction and electrostatic interaction.

### Effect of Cs^+^ concentration and adsorption isotherms

Adsorption isotherms are helpful to provide some information to describe the surface properties and the mechanism by which the interaction between the adsorbents and adsorbate^36^. Therefore, the adsorption capacities of GAPP were obtained by using different initial Cs^+^ concentrations of solution (50~600 mg/L), and the equilibrium data were fitted using Langmuir and Freundlich isotherm models (further description see Supporting Information, S1.1)^36,37^.

The experimental adsorption capacity of GAPP with different initial Cs^+^ concentrations as well as the calculated curves are presented in Fig. 4c. It was found that the correlation coefficient of Langmuir isotherm model (*R*^2^ = 0.9899) was bigger than that of Freundlich isotherm model (*R*^2^ = 0.9098), meaning the adsorption mechanism was better described by the Langmuir isotherm model and the monolayer adsorption in the uniform surface of GAPP. In addition, the value of *n* in Freundlich isotherm model was 2.13, which further suggested the favorable adsorption condition of Cs^+^ on GAPP^34,38^.

### Effect of adsorption time and adsorption kinetics

The Cs^+^ adsorption capacities *vs*. adsorption time were investigated with initial Cs^+^ concentration of 100 mg/L, which are shown in Fig. 4d. The adsorption capacity increased rapidly because of the sufficient available binding sites of GAPP at the beginning, and then tended to be slower with the decreasing of the available GAPP binding sites and the Cs^+^ concentration, and finally reached equilibrium within 24 h.

In this work, pseudo-first-order kinetic and pseudo-second-order kinetic model (S1.2) were used^39,40^, and the experiment data and the fitting curves are shown in Fig. 4d. It was found that the adsorption process of Cs^+^ on GAPP was better fitted by the pseudo-second-order model (*R*^2^ = 0.9882) than pseudo-first-order model (*R*^2^ = 0.9672), suggesting the major adsorption mechanism was chemisorption, and chemisorption was the rate-determining step, which was consistent with previous reports^22,23^.

### Effect of temperature and thermodynamics calculations

**Figure 5:**
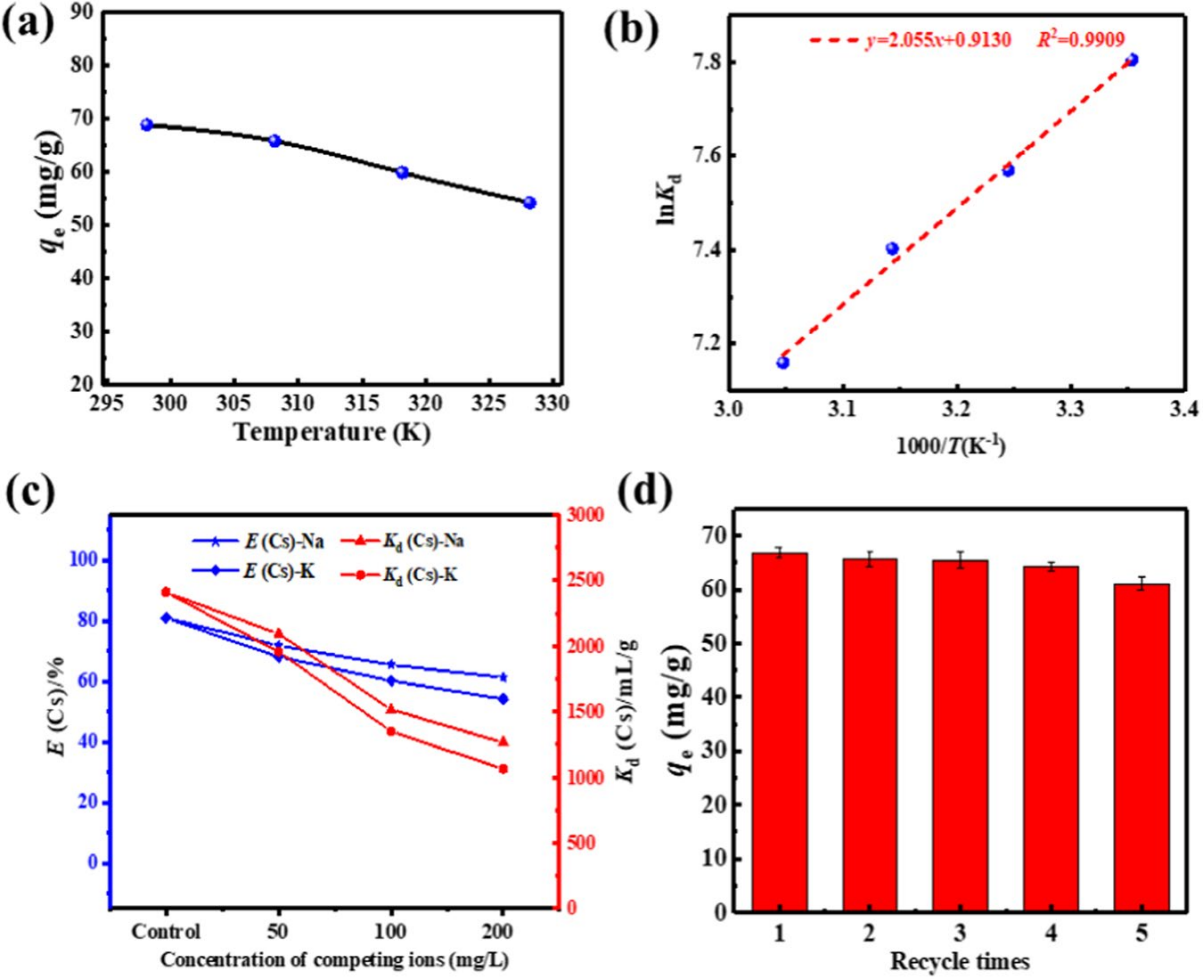
(**a**) Effect of temperature on Cs^+^ adsorption (*C*_0_ = 100 mg/L, *m*/*V* = 1 g/L, pH = 6.0, *t* = 24 h). (**b**) The Van’t Hoff plot; (**c**) Effect of concentration of competing ions on Cs^+^ adsorption (*C*^Cs^_0_ = 100 mg/L, *C*^Me^_0_ = 0–200 mg/L, *m*/*V* = 1 g/L, pH = 6.0, *t* = 24 h); (**d**) The recyclability of GAPP during consecutive adsorption-desorption process (adsorption: *C*_0_ = 100 mg/L, *m*/*V* = 1 g/L, pH = 6.0, *T* = 298.15 K, *t* = 24 h; desorption: 1 mol/L NH_4_Cl, *m*/*V* = 1 g/L, *T* = 298.15 K, 24 h).

To figure out the effect of temperature on Cs^+^ adsorption by GAPP, we carried out batch experiments under following conditions: initial Cs^+^ concentration of 100 mg/L, pH value of 6.0 and temperature in the range of 298.15–328.15 K. In Fig. 5a, the *q*_e_ decreased slowly with the increasing of temperature, which suggested the exothermic nature of the adsorption process.

The correlation between ln*K*_d_ and 1/*T* would provide essential information about the calculation of Δ*H*^*0*^ and Δ*S*^*0*^ in the adsorption process^41^. According to Fig. 5b, the values of Δ*H*^*0*^ and Δ*S*^*0*^ were calculated to be −17.08 kJ·mol^−1^ and 7.591 J·mol^−1^·K^−1^, respectively. Meanwhile, the values of Δ*G*^*0*^ at different temperatures were obtained and given in Table S1. The negative value of Δ*H*^*0*^ suggested the adsorption process belonged to exothermic process. In addition, the negative values of all the Δ*G*^*0*^ at temperature ranging from 298.15–328.15 K indicated the cesium adsorption into GAPP was spontaneous and feasible, which further suggested the affinity of GAPP on cesium.

### Effect of competitive cations on Cs^+^ adsorption

The selectivity of GAPP is of significance in terms of the co-existing metal ions in wastewater, especially alkaline metal ions Na^+^ and K^+^, which are the most ubiquitous and similar elements with Cs^+^. Therefore, the adsorption efficiency and distribution coefficient were determined in the presence of Na^+^ and K^+^ with different concentrations. The results are demonstrated in Fig. 5c, the adsorption efficiency and distribution coefficient gradually decreased with the increasing concentration Na^+^ and K^+^, and K^+^ could retard the selective adsorption of Cs^+^ on GAPP more seriously due to the closer similarity in hydration radius and chemical properties. However, the adsorption efficiency was about 20% less than that in pure Cs^+^ solution, and the distribution coefficient was larger than 1000 mL/g at even high concentrations of competitive Na^+^ or K^+^ ions. Therefore, GAPP could be accepted as a selective adsorbent for Cs^+^ adsorption.

### Selective adsorption of Cs^+^ from ground water

Considering that cesium generally enters water body from soil through different means, and to further evaluate the adsorption selectivity of GAPP over Cs^+^, selective experiments were studied using ground water, whose compositions of main metal cations, adsorption efficiency and distribution coefficients are presented in Table 1. The Cs^+^ adsorption efficiency could reach 99.5% and those for other metal cations were less than 16.8% after a simple absorption process. The distribution coefficient for Cs^+^ was about 2 × 10^5^ mL/g, which was much larger than some existing Cs^+^ adsorbents. As a result, the separation factors *S*_F_ of Cs^+^ over Na^+^, K^+^ and Ca^2+^ were 3485.1, 1297.3 and 985.6, respectively. The results above further suggested that GAPP showed an efficient adsorption performance and selectivity for Cs^+^ over Na^+^, K^+^ from ground water with complex competitive cations. Therefore, this GAPP could be accepted as a selective material with potential use on Cs^+^ removal from radioactive wastewater in presence of competitive cations.

**Table 1:** Concentrations of main metal cations in ground water and adsorption performance^a^.

	main metal cations				
	Cs^+^	Na^+^	K^+^	Ca^2+^	Mg^2+^
Concentration (mg/L)	17.58	682.01	137.90	211.05	—
*E*(%)	99.5	5.4	13.3	16.8	—
*K*_d_ (mL/g)	199000.2	57.1	153.4	201.9	—
*S*_*F*_	1	3485.1	1297.3	985.6	—

^a^GAPP dosage: 1 g/L, pH: 6.0, 298.15 K, 24 h.

### Desorption and reusability

To investigate the recycle performance of GAPP, the consecutive adsorption-desorption processes were carried out using NH_4_Cl solution as the eluent. As Fig. 5d shows, the adsorption capacity decreased slightly from 66.9 mg/g to 61.1 mg/g (decreased for about 8.6%) after being reused five times, indicating that the stable performance of GAPP in consecutive adsorption-desorption experiments. Furthermore, FT-IR and EDX analysis of GAPP after five-time recycle were carried out to demonstrate its stability during adsorption and desorption processes. To our delight, it was found that the FT-IR spectrum after adsorption (as Fig. S1b) was similar with that of original adsorbent (Fig. S1a) and further EDX analysis in Table S2 also indicated the favorable stability of GAPP during adsorption and desorption processes.

### Comparison of GAPP with other adsorbents

A comparison of GAPP and other materials was conducted to assess the potential application of GAPP From Table 2, we could find that the obtained adsorption capacity of GAPP (163.6 mg/g) on Cs^+^ was much larger than some biomass-based adsorbents, such as CPW gel (71.8 mg/g), SSM (52.4 mg/g), Bn-CTS (57.1 mg/g), MgP-MS (64.0 mg/g), and comparable with CPT gel (178.2 mg/g) and CPW gel (162.3 mg/g). However, CPT gel and CPW gel could only be effective at a narrow pH rang (3–6), and they could not be recycled and reused. Compared with other inorganic adsorbents, the selectivity and adsorption capacity of GAPP was also outstanding at a broad pH range (2–12). Furthermore, it was selective over alkaline metals Na^+^ and K^+^ in ground water and shows stable performance during consecutive adsorption-desorption experiments. Therefore, we believe this cost-effective, robust and efficient adsorbent can be accepted as an effective material with potential application in Cs^+^ removal from radioactive wastewater.

**Table 2:** Comparison of various Cs^+^ adsorbents in the literature and GAPP.

Materials	Adsorption capacity (mg/g)	Active pH range^*a*^	Selectivity	Reusability	Ref.
CPT gel	178.2	2.5–6.5	*vs*. Na^+^,	Limited	^22^
CTL gel	162.3	3–6	*vs*. Na^+^	Limited	^22^
CPW gel	71.8	2.5–7.5	—	Limited	^23^
SSM	52.4	2–12	*vs*. Na^+^, K^+^	Reusable	^42^
Bn-CTS	57.1	3–10	*vs*. Li^+^, Na^+^, K^+^, Mg^2+^	Reusable	^43^
MgP-MS	64.0	5–10	—	Reusable	^44^
Magnetic 4 A zeolite	106.6	—	—	—	^45^
Fe_3_O_4_-O-CMK-3	205	3–11	*vs*. Li^+^, Na^+^, K^+^, Ca^2+^, Sr^2+^	Reusable	^46^
mag-AMP	83.33	2–12	—	Reusable	^41^
MMT-PB	57.47	—	*vs*. Na^+^, K^+^, Mg^2+^, Ca^2+^	—	^47^
**GAPP**	**163.6**	**2–12**	***vs****.* **Na**^**+**^**, K**^**+**^	**Reusable**	**this work**

^*a*^pH range in which the adsorbents could maintain more than 50% of the maximum adsorption capacity^48,49^.

## Conclusions

In this work, cost-effective and robust porous polymers were fabricated using biomass polyphenol sources *via* a facile azo-coupling method and applied for Cs^+^ separation from aqueous solutions. Due to the porous structures functionalized by biomass polyphenols, the gallic acid-based porous polymer (GAPP) exhibited stable performance at wide pH value range with a maximum adsorption capacity of 163.6 mg/g, placing it at top material for Cs^+^ adsorption. More importantly, GAPP showed significantly high adsorption performance toward Cs^+^ compared to Na^+^ and K^+^, making it possible in selective removal of Cs^+^ from ground water in presence of co-existing competitive ions. In addition, the Cs-laden GAPP could be facilely eluted and reused in consecutive adsorption-desorption process. Therefore, we believe that this low-cost, robust and selective GAPP is a promising material with potential application on ^137^Cs removal from radioactive wastewater, and we hope this work could provide more ideas about the potential utilization of biomass polyphenols in representative fields.

## Materials and Methods

### Chemicals and materials

Gallic acid (GA, 98%), tannic acid (TA, 95%), 1,3,5-trihydroxybenzene (phloroglucinol, HB, 99%) were supplied by J & K Scientific Ltd. Ellagic acid (EA, 98%) and arbutin (AR, 98%) were obtained from Macklin Biochemical Co., Ltd. Benzidine (BE, 98%) was obtained from Aladdin. Cesium chloride, sodium nitrite, sodium carbonate and other regents were provided by Tianjin Guangfu Reagent. Ground water sampled from Tibet, China was used for adsorption performance evaluation of the adsorbents.

### Preparation of porous polymers

**Scheme 2:**
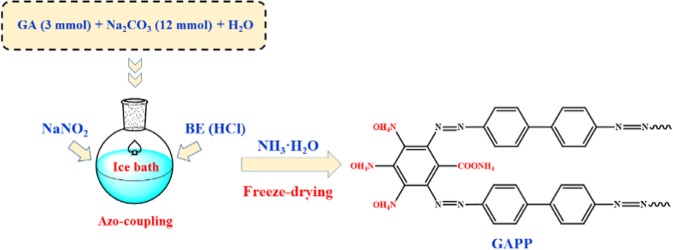
Preparation roadmap of GA.

The GAPP was prepared using gallic acid as starting material, and the synthesis procedure was shown as Scheme 2 for example, and those for other PPs were similar. BE (5 mmol) was firstly dissolved in 5% HCl solution, and NaNO_2_ solution (10 mmol) was then added and stirred for 30 min. After that, the solution was contacted with a mixture of GA (3 mmol) and Na_2_CO_3_ (12 mmol) at ice-water mixture. After reaction for 12 h, the mixtures were separated by simple centrifugation for 30 min (Z326, Germany) and washed by H_2_O and ethanol. Subsequently, it was subjected to NH_3_·H_2_O solution for about 12 h. Finally, it was washed by water for three times and freeze-drying for further use.

### Instrumentation

FT-IR spectroscopy (Tensor 27, Germany) was used to identify the groups in the materials. Powder X-ray diffraction (XRD) were collected on X-ray diffractometer with Cu Kα radiation (MSAL XD-3, China). The thermal property of GAPP was evaluated using a thermogravimetric analysis (TGA) instrument (Seteram Labsys, France) over the temperature range of 30–800 °C under Ar atmosphere at 10 °C/min heating rate. The surface properties of GAPP was characterized by SEM (JSM-IT300LV, Japan). The transmission electron microscopy (TEM) image was determined by a TEM JEM-1011. The elemental compositions were determined by EDX (X-Max 20, Oxford Instruments). The N_2_ adsorption-desorption isotherms were obtained using a physical adsorption analyzer (ASAP 2020, Micromeritics) and the surface area was obtained by the BET method.

### Batch experiments

In each experiment, the desired amounts of adsorbents and Cs^+^ solution with given initial concentrations were loaded in a polytetrafluoroethylene bottle, and then it was shaken in a thermostat with certain temperature. After that, it was stopped and centrifuged to take the clarified supernatant for chemical analysis. The concentrations of metallic ions were determined by ICP-OES. Moreover, the detailed experimental conditions were described in the figures and tables captions. The key parameters during the adsorption, such as adsorption efficiency (*E*, %), adsorption capacity (*q*_t_, mg/g), distribution coefficient (*K*_d_, mL/g) and separation factor (*S*_F_) were calculated by Eqs. 1–4.

1 $$E( \% )=\frac{{C}_{0}-{C}_{t}}{{C}_{0}}\times 100$$

2 $${q}_{t}({\rm{mg}}/{\rm{g}})=\frac{({C}_{0}-{C}_{t})V}{m}$$

3 $$K{\rm{d}}\,({\rm{mL}}/{\rm{g}})=\frac{(C0-Ce)}{Ce}\times \frac{1000V}{m}$$

4 $${S}_{{\rm{F}}}=\frac{K{{\rm{d}}}^{{\rm{Cs}}}}{K{{\rm{d}}}^{{\rm{Me}}}}$$

where *C*_*0*_, *C*_*t*_ and *C*_*e*_ (mg/L) represent the original, final and the equilibrium concentrations of Cs^+^; *V* (L) refers to the aqueous solution volume; *m* (g) represents the mass of the adsorbent; Me are different competitive cations.

## Supplementary information

Supplementary information.
