# Supplementary material for: Facile Synthesis of Porous Polymer Using Biomass Polyphenol Source for Highly Efficient Separation of Cs+ from Aqueous Solution
Source: Sci Rep. 2020 May 19;10:8221. doi: 10.1038/s41598-020-65099-6 (PMC7237466; doi:10.1038/s41598-020-65099-6)
Supplement: Supplementary file 1 — Supplementary information. [file 41598_2020_65099_MOESM1_ESM.docx]

Supporting Information

**Facile synthesis of porous polymer using biomass polyphenol source for highly efficient separation of Cs^+^ from aqueous solution**

Shangqing Chen, Jiayin Hu^*^, Yafei Guo, Tianlong Deng^*^

Tianjin Key Laboratory of Brine Chemical Engineering and Resource Eco-utilization, College of Chemical Engineering and Materials Science, Tianjin University of Science and Technology, Tianjin, 300457, PR China

***Corresponding Author**

Tel. and Fax: 86-22-60601156

E-mail: [hujiayin@tust.edu.cn](mailto:hujiayin@tust.edu.cn) and [tldeng@tust.edu.cn](mailto:tldeng@tust.edu.cn)

**Contents**

**S1. Adsorption models..………..………………………………………………..2**

**S2. Adsorbent stability…………………………………………………………..4**

**S1. Adsorption models**

**S1.1 Isotherm model**

For Langmuir isotherm model, the empirical equation is given as followed:^1^

$q_{e}=\frac{Q_{\max}K_{L}C_{e}}{1+K_{L}C_{e}}$ (1)

where *q*_e_ is the adsorption capacity at equilibrium (mg/g), *C*_e_ is the equilibrium concentration (mg/L). *Q*_max_ refers to the maximum adsorption capacity (mg/g), and *K_L_* is the adsorption intensity or Langmuir coefficient related to the affinity of the binding site (L/mg).

Freundlich empirical equation is expressed as:^2^

 (2)

where *K*_F_ (mg/g) and 1/*n* are the constants related to the adsorption capacity and the adsorption intensity, respectively.

**S1.2 Kinetic model**

In order to clarify the mass transfer reality of the adsorption process, the frequently-used pseudo-first-order kinetic and pseudo-second-order kinetic model^3,4^ were used to evaluate the effect of contact time on the adsorption capacity of GA-POP, respectively.

pseudo-first-order: $\ln(q_{\text{e}}-q_{\text{t}})=\ln q_{\text{e}}-k_{1}t$ (3)

pseudo-second-order: $\frac{t}{q_{t}}=(\frac{1}{k_{2}q_{\text{e}}^{2}})+\frac{t}{q_{\text{e}}}$ (4)

where *q*_e_ and *q*_t_ (mg/g) are the adsorption capacities at equilibrium and at time *t*; and *k*_1_ (min^-1^) and *k*_2_ (g·mg·min^-1^) denote the pseudo-first-order and pseudo-second-order constants.

**S1.3 Thermodynamic study**

Thermodynamic study is an essential part for adsorption, and thermodynamic parameters could provide further information on inherent changes during adsorption process. Therefore, studies concerning adsorption thermodynamics were performed and thermodynamics parameters including enthalpy (Δ*H^0^*, kJ·mol^-1^), entropy (Δ*S^0^*, J·mol^-1^·K^-1^) and Gibbs free energy (Δ*G^0^*, kJ·mol^-1^) were calculated by following equations:^5^

 (5)

$\Delta G^{0}=\Delta H^{0}-T\Delta S^{0}$ (6)

where *K*_d_ is the equilibrium distribution coefficient, *T*(K) is the temperature, *R* refers to the universal gas constant with the value of 8.314 J·mol^-1^·K^-1^, respectively.

**Table S1** Thermodynamic parameters of the adsorption process.

| *T*(K) | Δ*G*(kJ·mol^-1^) | Δ*H*(kJ·mol^-1^) | Δ*S*(J·mol^-1^·K^-1^) |
| --- | --- | --- | --- |
| 298.15 | -2.28 | -17.08 | 7.591 |
| 308.15 | -2.36 |  |  |
| 318.15 | -2.43 |  |  |
| 328.15 | -2.51 |  |  |

**S2. Adsorbents stability**

**Figure S1.** FT-IR spectra: a, GAPP before (a) and after (b) adsorption.

**Table S2.** EDX analysis of GAPP before and after adsorption.

| Component | Mass fraction (%) | |
| --- | --- | --- |
|  | before adsorption | after adsorption |
| Carbon (C) | 66.78 | 65.08 |
| Oxygen (O) | 24.69 | 22.00 |
| Nitrogen (N) | 8.53 | 7.83 |
| Cesium (Cs) | 0.00 | 5.09 |

**References**

[1] I. Langmuir, The adsorption of gases on plane surface of glass, mica and platinum. J. Am. Chem. Soc., 40 (1918) 1361-1403.

[2] H. M. F. Freundlich, Über die adsorption in Lösungen. Z. Phys. Chem. 57A (1906) 385-470.

[3] P.A. Kumar, M. Ray, S. Chakraborty, Adsorption behaviour of trivalent chromium on amine-based polymer aniline formaldehyde condensate. Chem. Eng. J., 149 (2009) 340-347.

[4] T. Nur, P. Loganathan, J. Kandasamy, S. Vigneswaran, Removal of strontium from aqueous solutions and synthetic seawater using resorcinol formaldehyde polycondensate resin, Desalination, 420 (2017) 283-291.

[5] H.M. Yang, K.S. Hwang, C.W. Park, K.W. Lee, Sodium-copper hexacyanoferrate-functionalized magnetic nanoclusters for the highly efficient magnetic removal of radioactive caesium from seawater, Water Res., 125 (2017) 81-90.
